# 

**Published:** 1896-09

**Authors:** 


					﻿ESTABLISHED 1855.
SUBSCRIPTION, $2.00.
ATLANTA
MEDICAL AND SURGICAL
JOURNAL.
new: semes.’ Atlanta, Ga., September, 1896.	N®. 7.'
				

